# SARS-CoV-2 Positivity and Mask Utilization Among Health Care Workers

**DOI:** 10.1001/jamanetworkopen.2021.14325

**Published:** 2021-06-22

**Authors:** Aldon Li, Jeff Slezak, Ana Miranda Maldonado, June Concepcion, Catherine Voloso Maier, Gunter Rieg

**Affiliations:** 1 Permanente Medicine, Southern California Permanente Medical Group, Pasadena; 2 University of California, Riverside School of Medicine, Riverside; 3 Kaiser Permanente Department of Research and Evaluation, Pasadena, California; 4 Kaiser Permanente Southern California, Pasadena; 5 Kaiser Permanente Bernard J. Tyson School of Medicine, Pasadena, California; 6 David Geffen School of Medicine at UCLA, Los Angeles, California

## Abstract

This cohort study examines the association of mask use with COVID-19 transmission among health care workers in California.

## Introduction

Human-to-human transmission of SARS-CoV-2 occurs during exposure to infectious respiratory droplets or aerosols generated by humans with COVID-19.^1^ Aerosolizing events (AEs) contribute to the controversy regarding the selection of optimal personal protective equipment (PPE) for preventing transmission of SARS-CoV-2 to health care workers (HCWs). Because of global PPE shortages,^2^ further studies examining the association of HCW PPE use with the acquisition of COVID-19 are needed to protect our HCWs and decrease inappropriate PPE use.

## Methods

We conducted a retrospective cohort study in a single service area at Kaiser Permanente Southern California (KPSC), identifying all HCWs who underwent testing for COVID-19 by polymerase chain reaction during March 13 through August 3, 2020. HCWs were identified for testing either through (1) exposure to a patient with COVID-19 or (2) symptomatology of potential COVID-19 as defined by US Centers for Disease and Control and Prevention (CDC) criteria.^3^ The exposure definition at our institution aligned with CDC criteria.^4^

The KPSC institutional review board approved this study and waived informed consent because data were deidentified and involved no more than minimal risk to participants. We followed the Strengthening the Reporting of Observational Studies in Epidemiology (STROBE) reporting guideline for cohort studies.

Contact tracing by structured interview was conducted with all HCWs who underwent testing. This process consisted of documentation of exposure status, masking protocol compliance, and testing results.

Masking protocols for HCWs caring for patients with confirmed or suspected COVID-19 consist of medical masks (MMs) when performing nonaerosolizing, routine patient care and respirator masks (RMs) for patient care in areas with high risk of AEs, identified as emergency, urgent care, and designated COVID-19 medical and surgical units and intensive care units. Patient care not related to COVID-19 and non–patient care encounters did not require any PPE until universal masking was implemented.

The testing protocol for exposed HCWs was initiated on the day when exposure was identified, then again 5 to 7 days after exposure, and finally at day 14 after exposure. A symptomatic HCW was tested on the first day of reported symptoms. Logistic regression was used to assess the association between mask usage and test positivity. Analysis was performed using SAS software version 9.4 (SAS Institute) with 2-tailed testing and *P* < .05 as the statistical significance level.

## Results

Overall, 1414 HCWs were tested for SARS-CoV-2, with an overall positivity rate of 6.7% (95 HCWs) compared with KPSC community patient positivity rate of 13.9% during the study period. Of the 95 HCWs with positive test results, 91 (95.8%) acquired COVID-19 outside of a known patient-related exposure event.

Of the 1414 HCW, 595 (42.1%) had exposures defined by CDC criteria, 961 (68.0%) developed symptoms, 438 (31.0%) worked in high-risk areas, and 396 (28.0%) had underlying health conditions. Furthermore, 302 (21.4%) wore RM, 724 (51.2%) wore MM, and 388 (27.4%) wore no masks (NM). In unadjusted analysis, we found no difference in HCW positivity between HCW wearing RM vs MM (Table), but a statistically significant lower positivity was found when HCW wore RM vs NM (odds ratio, 0.50; 95% CI, 0.28-0.90; *P* = .02) and MM vs NM (odds ratio, 0.45; 95% CI, 0.29-0.72; *P* < .001). We found a similar association between mask use and test positivity after adjusting for confounders (Table).

**Table.** SARS-CoV-2 Positivity in 1414 HCWs Using MM Compared With RM or NM From March 13 to August 3, 2020^a^

Mask use	HCWs undergoing SARS-CoV-2 testing, No. (%)		Odds ratio (95% CI)		*P* value
	Positive (n = 95)^b^	Negative (n = 1319)	Unadjusted	Adjusted^c^	
RM, ie, N95 or higher-level respirators (n = 302)	17 (5.6)	285 (94.4)			
Exposed	0	114 (40.0)	1.11 (0.61-2.00)	1.23 (0.66-2.29)	.51
Symptomatic	16 (94.1)	142 (49.8)			
Exposed with symptoms	1 (5.9)	29 (10.2)			
MM, ie, surgical or procedural mask (n = 724)	37 (5.1)	687 (94.9)			
Exposed	0	229 (33.3)	1 [Reference]	1 [Reference]	NA
Symptomatic	36 (97.3)	389 (56.6)			
Exposed with symptoms	1 (2.7)	69 (10.4)			
NM (n = 388)	41 (10.6)	347 (89.4)			
Exposed	1 (2.4)	109 (31.4)	2.92 (1.38-3.48)	2.12 (1.31-3.41)	.002
Symptomatic	39 (95.1)	197 (56.8)			
Exposed with symptoms	1 (2.4)	41 (11.8)			

Abbreviations: HCW, health care worker; MM, medical mask; NA, not applicable; NM, no mask; RM, respirator mask.

^a^ Total HCW occupation breakdown is as follows: 565 (40.0%), nursing (registered nurse, certified nursing assistant, licensed vocational nurse, medical assistant, or nurse practitioner); 141 (10.0%), physicians (MD and DO); and 708 (50.0%), other HCWs (technician, clerk, nutrition, environmental services, laboratory, or transport).

^b^ Positive test occupation breakdown is as follows: 42 (44.2%), nursing (registered nurse, certified nursing assistant, licensed vocational nurse, medical assistant, or nurse practitioner); 3 (3.2%), physicians (MD or DO); 50 (52.6%), other HCWs (technician, clerk, environmental services, laboratory, or transport).

^c^ Adjusted for HCW exposure status, presence of symptoms, presence of underlying health conditions, and work location in risk-areas.

## Discussion

Our study found no association in positivity rates among HCW wearing RM vs MM when performing nonaerosolizing, routine patient care, supporting findings from a recent case-control study^5^ and an earlier case report^6^ suggesting that MM protected HCWs from acquiring COVID-19. Only 4 positive tests followed exposure events (2 among nursing staff, 2 among technician staff), none were from confirmed AEs, and all occurred prior to universal masking implementation. More than 95% of HCWs acquired COVID-19 outside of a known patient-related exposure event, possibly due to improper donning and doffing of masks during social interactions with other HCWs in the workplace or in the community, supporting a study^5^ finding higher risk of COVID-19 transmission outside of patient care interactions.

Our low exposure conversion may reflect the low incidence of AEs in our data set and early adoption of universal masking. These factors may have decreased high-risk exposures of unmasked HCWs to unmasked infectious patients.

Strengths of our study include delineating a large cohort with complete individual-level masking and testing data to quantify risk of COVID-19 in the health care setting. Limitations of our study include the potential for recall bias during contact tracing interviews, the unavailability of whole genome sequencing at our institution to confirm the transmission event, and the retrospective nature of the study design. Randomized studies in mask optimization would be ideal, although they may face challenges in enrollment.
